# Patient experience of bladder cancer: A data linkage study

**DOI:** 10.1002/bco2.325

**Published:** 2024-01-31

**Authors:** Snehadhar Shah, Jonathan Ince, Roger Kockelbergh

**Affiliations:** ^1^ University Hospitals of Leicester NHS Trust Leicester UK; ^2^ University of Leicester Leicester UK

**Keywords:** activities of daily living, bladder cancer, body image, patient experience, psychological impact, quality of life, symptoms

## Abstract

**Introduction:**

Bladder cancer is one of the most common cancers worldwide and can be managed with a range of approaches, including conservative, medical and surgical therapies. Treatment may be associated with considerable morbidity, but despite this, little data exist to reflect patients' subsequent experience. This study aims to evaluate patients' experiences of bladder cancer care by linking data from a national cancer experience survey with data routinely collected from National Health Service (NHS) sources. This study considers patient perspectives and makes recommendations to improve the patient experience of bladder cancer care.

**Methods:**

Anonymised data from the National Cancer Patient Experience Survey (NCPES) for patients who had received care for bladder cancer were collated and linked with demographic and treatment data. Questions from the NCPES were then categorised into different themes based on their content. This study focused on themes relating to lifestyle, activities of daily living (ADL), symptoms, psychological impact and body perception. Statistical analyses were used to investigate the relationship between patient reported experience, demographics and type of care received.

**Results:**

NCPES data from 673 patients (487 male, 29 undisclosed) with at least T1 bladder cancer were analysed. Statistically significant differences were identified across the five investigated patient experience themes. No significant difference was seen in patient reported experience between bladder cancer drug treatments (such as intravesical BCG vs. intravesical chemotherapy vs. systemic chemotherapy) and radiotherapy types (curative vs. palliative). Patients treated with cystectomy had significantly worse experiences relating to body image and ADL but not when compared with patients treated with radical radiotherapy. Patients with long‐term health conditions reported worse experiences for all five themes compared with those without.

**Conclusion:**

The literature surrounding the experience of patients with bladder cancer is limited. This data linkage study demonstrates the impact of bladder cancer care on five patient experience themes, including the effect of different treatment types and the presence of long‐term health conditions. While limited by sample size and data comprehensiveness, this study aims to inform clinicians and service providers of factors affecting patient experience of bladder cancer care, to stimulate service review and development.

## 1 INTRODUCTION

Bladder cancer is the tenth most common cancer worldwide, with an increasing incidence in some developed countries.^1^ The most common type of bladder cancer is urothelial, arising from the transitional cells, and forms up to 90% of diagnoses.^1^ Smoking and occupational exposure are risk factors for bladder cancer, a factor contributing to the up to 4‐fold relative risk in males compared with females.^1^

Management of bladder cancer is not uniform and is selected by factors including patient demographics, tumour characteristics and the goal of therapy (such as curative or palliative). Management strategies typically include endoscopic resection, radiotherapy, chemotherapy, immunotherapy and radical surgical approaches such as cystectomy.^2^ Side effects are related to treatment selection ranging from adverse reactions to chemotherapy and radiotherapy, to intra‐ and post‐operative complications associated with surgery.^3^, ^4^

Despite the high incidence, relatively little is known about patient experience of bladder cancer, as highlighted in a recent systematic review.^5^ However, this information is important when helping patients to select management options and should also guide improvements to patient care pathways.

In 2017, Edmondson et al. performed a systematic review investigating the lived experiences of patients with bladder cancer.^5^ The review highlighted the effect of bladder cancer and the associated therapies on patient's lives, from diagnosis to post‐treatment. This included a study by Cerruto et al. that considered the changes in quality of life (QOL) for patients who underwent a radical cystectomy, and how this changed with time.^6^ The review demonstrated a paucity of patient experience evidence for bladder cancer. Significant findings were found relating to treatment decision making, post‐treatment complications and impacts on different domains to patients' lives (such as body image and sexuality).^5^ Recent data from Tan et al.^7^ used a mixed methods approach in a subset of patients from the DETECT II trial with a new diagnosis of bladder cancer. Findings included limited awareness, a period of increased anxiety following initial cystoscopy while awaiting TURBT and a concern that their disease would have a prolonged trajectory.

The burdens associated with bladder cancer and its management were reported by Mohamed et al. who performed qualitative interviews for patients with muscle‐invasive bladder cancer.^8^ Key findings included unmet needs throughout the patient journey, a lack of understanding about treatment options and difficulty managing in the ‘survivorship phase’ following treatment. Some patients reported depression, worries about cancer recurrence, and challenges when returning to previous physical or social activities.^8^

Physical, sleep and rest aspects of life may be adversely affected in patients with muscle‐invasive bladder cancer.^9^ A 2022 study found that physical aspects to QOL were significantly impacted in older patients, those with other malignancies, cardiovascular comorbidity or advanced disease.^9^ The study also found that patients who had a radical cystectomy reported improved sleep and rest compared with those with bladder sparing therapy.^9^

A large cross‐sectional study assessing QOL for patients after bladder cancer found there was no significant difference in reported QOL domains between cancer stages and treatment groups (once age‐sex adjusted).^10^ However, it was noted that impacts on QOL were greater for patients with more than four long‐term conditions (compared with those with none), and with increasing age.^10^ The study also identified impacts on QOL domains relating to sexual function and financial difficulties.^10^ Finally, the cross‐sectional study found that when compared with the general population or to patients who had prostate or colorectal cancers, patients with bladder cancer reported more problems across all QOL domains, with the exception of anxiety and depression.^10^

In the United Kingdom, the National Health Service (NHS) produces a cancer experience survey periodically, which is based on the experience of patients who have undergone treatment for cancer (the National Cancer Patient Experience Survey, NCPES).^11^ The survey covers multiple aspects of life and across the timeline of disease management, with the aim of monitoring national cancer statistics and improving quality of care at local levels.

This study evaluates the data from the NCPES for patients diagnosed with bladder cancer, with the aim of exploring the effects of patient factors and treatment type on patient experiences measures. Findings from this study offer a detailed insight into the patient experience, supporting future care and the design of service provision.

## 2 METHODS

Data was obtained from the National Cancer Registration and Analysis Service (NCRAS) through the Office for Data Release (ODR). Individual patient data from the following datasets were linked by NHS number and anonymised by NCRAS: NCPES, Hospital Episode Statistics (HES), National Radiotherapy dataset (RTDS) and the Systemic Anti‐Cancer Therapy dataset (SACT).

The dataset included individual responses from patients with bladder cancer who answered the 2015 NCPES,^11^ with data including patient demographics, bladder cancer management type and frequency, and the NCPES questionnaire answers. The 2015 NCPES was selected to allow a sufficient follow‐up period for the other datasets. Treatment location was not released by the ODR to preserve data anonymisation. The dataset was reviewed and duplicate entries were removed. In instances of multiple responses from a single participant, only the most complete entry was kept.

Given the multiple data sources, missing data were frequently seen for patients, including where demographic information was missing or where patients had not answered individual NCPES questions. In this case, patients were not excluded but were only included in the individual analyses where data was sufficient.

For each question, a score was given based on the participant's response. For example, if a question had five answer options ranging from ‘low impact’ to ‘high impact’, a score of 5 would be given to the ‘high impact’ and a score of 1 for ‘low impact’.

Given the broad nature of the included questions in the NCPES, the 99 questions were organised into broad themes by two of the co‐authors (S. S. and J. I.). These themes were based on common trends seen within the questions. The most populated themes included: lifestyle, activities of daily living (ADL), symptoms, psychology and body perception. Some questions did not fit within these themes, but due to the low number of outlier questions, new themes could not be created to accommodate them, so those questions were not included in further analysis.

The lifestyle theme included questions relating to patient's day‐to‐day lifestyle, including questions linked to content and enjoyment of life, sleep quality and ability to take holidays. The ADL theme focused on questions related to self‐care, independence, and difficulties in carrying out day‐to‐day activities. Questions relating to sexual, urinary and bowel symptoms were grouped in the symptom theme. The psychology theme included questions asking about anxiety, depression, emotional support, mental health and thoughts/fears regarding cancer and death. Finally, the body perception theme linked questions asking about thoughts on weight, appetite, body image and how individuals felt about their stoma.

Using these themes, each participant had a total score calculated for each theme, based on their NCPES responses. These scores were reported as a proportion of the maximum possible score for each theme, with a score of 1.0 suggesting maximum impact on that element of the patient's life.

For further comparisons to be made, each respondent was organised into a banding of a ‘low’, ‘medium’ or ‘high’ score. Bandings were one third of the range between minimum and maximum possible theme scores.

Data were then imported into Stata (StataCorp) for statistical analyses comparing the five theme scores with patient demographics and bladder cancer management type. Bladder cancer management types described were cystectomy, transurethral resection of a bladder tumour (TURBT), radiotherapy (curative or palliative intent) and chemotherapy type (including Bacillus Calmette–Guerin [BCG], intravesical or systemic).

Shapiro–Wilk tests were used to assess data normality; as comparator groups were non‐normally distributed, non‐parametric tests were used (Mann–Whitney and Kruskal–Wallis tests). *p* value <0.05 was considered statistically significant.

## 3 RESULTS

Data from 673 patients with at least T1 bladder cancer (487 male, 29 undisclosed) who responded to the NCPES were analysed in this study. The most common cancer histology was transitional cell carcinoma (92%), 67% of patients had Grade 3 malignancy.

Demographics of patients included in the study are summarised in Table 1. Patients were organised into age range bandings, with the largest group being patients aged between 70 and 74 years (18.9%). Smoking history demonstrated that 9.8% of patients were current smokers while 51.2% were ex‐smokers. Sixty two percent of patients reported having a long‐term health condition and 22.6% reported having a carer role.

**TABLE 1:** Demographics of patients included within the study.

Characteristic	Description of sample	
Sex		
Male	487	75.6%
Female	157	24.4%
Age (years)		
Under 60	85	13.0%
60–64	89	13.6%
65–69	121	18.5%
70–74	124	18.9%
75–79	111	17.0%
80–84	76	11.6%
85+	49	7.5%
Smoking status		
Smoker	63	9.8%
Ex‐smoker	328	51.2%
Non‐smoker	250	39.0%
Other long‐term health conditions		
Yes	376	62.0%
No	203	33.5%
Do not know/cannot say	27	4.5%
Performs a caring role		
Yes	138	22.6%
No	473	77.4%
Treatment type		
Chemotherapy	145	21.7%
Cystectomy	110	17.6%
Radiotherapy	77	—^b^
TURBT	609	97.3%
Chemotherapy type		
BCG	22	16.9%
Intravesical	9	6.9%
Systemic	99	76.2%
Radiotherapy intent		
Curative	58	75.3%
Palliative	19^a^	24.7%
Number of TURBTs		
One	314	51.6%
Two	210	34.5%
Three	62	10.2%
Four	20	3.3%
Five	3	0.5%
Has a stoma		
Yes	96	19.8%
No	390	80.2%

*Note*: If a patient did not answer the characteristic question, they were not included in the sample description.

^a^ Note that 10 patients were found to initially receive curative therapy.

^b^ Percentage not calculated as total number of respondents not noted.

TURBT was the most common procedure (609 patients, 97.3%), approximately half of patients (51.6%) reported only one TURBT. Anticancer drug therapy was reported for 145 patients (21.7%), systemic chemotherapy represented the majority (99, 76.2%) while BCG was used in 22 (15%). Radiotherapy was less frequently reported (77 patients), 75.3% reported curative intent and 24.7% a palliative approach. Of the 19 patients receiving palliative radiotherapy, 10 had previous radical radiotherapy. Cystectomy was reported in 110 patients (17.6%), the least common treatment. In addition, 108 patients had stoma and only 2 had a continent urinary diversion; hence, it was not possible to compare QOL for continent versus incontinent diversion.

### 3.1 Lifestyle impact

No significant differences in lifestyle impact were identified between sex, age category and management type. However, the impact on lifestyle was significantly worse for patients who smoke, with respective medians [IQR] between smokers, ex‐smokers and non‐smokers of 0.46 [0.33, 0.59], 0.44 [0.35, 0.56], 0.41 [0.33, 0.51], *p* = 0.021. Patients with long‐term health conditions also reported significantly worse impact on their lifestyle, with a median impact score of 0.47 [0.38, 0.57] compared with 0.39 [0.30, 0.46] for those without long‐term health conditions (*p* < 0.001). Lifestyle impact was significantly worse for patients who reported having a carer role, with an overall impact score of 0.46 [0.37, 0.57] compared with 0.42 [0.33, 0.42] of those without a caring role (*p* = 0.004).

### 3.2 ADL impact

Significant differences in impact on ADLs were identified with increasing age, those who had cystectomies, those with long‐term health conditions, and patients with a caring role. No significant differences were identified across other demographics.

Increasing age was associated with increased impact on ADLs. Median scores were similar between under 60 years (0.25 [0.24, 0.39]), 60–64 years (0.25 [0.24, 0.33]) and 65–69 years (0.25 [0.24, 0.35]). The remaining age groups (70–74, 75–80, 80–84 and over 85 years) had higher impact scores of 0.28 [0.24, 0.39], 0.29 [0.24, 0.40], 0.32 [0.25, 0.45] and 0.33 [0.26, 0.51] respectively, *p* = 0.001.

Like the QOL theme, patients with long‐term health conditions and carer roles were found to have significantly increased impact scores for ADL. Patients with long‐term conditions had a median impact score of 0.31 [0.24, 0.47] compared with 0.24 [0.24, 0.28] in those without (*p* < 0.001). Patients with carer roles had a median score of 0.29 [0.24, 0.42] compared with 0.26 [0.24, 0.37] in those without carer roles (*p* = 0.021).

Finally, patients who had a cystectomy (*n* = 110) reported a significantly larger impact on their ADLs compared with those who did not receive a cystectomy (*n* = 516). Respective median impact scores were 0.29 [0.25, 0.44] and 0.27 [0.24, 0.38], *p* = 0.028. No difference was seen when cystectomy was compared with radical radiotherapy.

### 3.3 Symptom impact

Significantly worse genitourinary symptom impact scores were identified in males, increased age and in those with long‐term health conditions. No significant differences were reported between treatment groups.

An increased impact of symptoms was reported in males, with a median impact score of 0.49 [0.41, 0.57] compared with 0.43 [0.36, 0.54] in females (*p* < 0.001).

Increased age was associated with an increased reported impact of symptoms (*p* = 0.012). Median impact scores were found to increase with increasing age category, with values of 0.45 [0.36, 0.54], 0.48 [0.41, 0.55], 0.47 [0.39, 0.55], 0.48 [0.39, 0.55], 0.50 [0.41, 0.58], 0.50 [0.42, 0.57] and 0.56 [0.43, 0.61], respectively.

Finally, patients with long‐term health conditions reported a worse impact from symptoms (0.50 [0.42, 0.57]) compared with those without long‐term health conditions (0.48 [0.36, 0.52]), *p* < 0.001.

### 3.4 Psychological impact

The impact of bladder cancer and its management on psychological factors (including mental health, emotional support and thoughts or fears relating to cancer and death) was only related to smoking status and the presence of a long‐term health condition.

Increased psychological impact scores were reported among smokers and ex‐smokers when compared with non‐smokers (*p* = 0.009). Respective median impacts scores were 0.47 [0.37, 0.56], 0.47 [0.38, 0.56] and 0.44 [0.35, 0.52].

Significantly increased psychological impact scores were also reported for patients with long‐term health conditions, 0.47 [0.37, 0.56] and 0.44 [0.35, 0.51], respectively (*p* = 0.006).

### 3.5 Body perception impact

Body perception impact scores for age, long‐term health conditions and having had a cystectomy were all found to show significant differences. As this theme included questions about stomas (where relevant), no direct comparisons could be made between scores and presence of a stoma.

Although a significant difference was seen between age categories (*p* = 0.004), no clear trend was seen in the median impact scores between age groups. Median values for the under 60, 60–64and 65–70 years were similarly high with the same medians of 0.50 [0.38, 0.63], while values were lower for the 70–74 and 75–80 years (0.38 [0.25, 0.63] and 0.38 [0.33, 0.50], respectively). Median impact score for 80–84 years increased to 0.50 [0.38, 0.63] but returned to 0.38 [0.25, 0.63] for over 85 years.

As with the other themes, presence of a long‐term health condition was also associated with an increased body perception score, with a median score of 0.50 [0.38, 0.63] for those with a long‐term health condition compared with 0.38 [0.25, 0.63] for those without (*p* = 0.010).

Finally, a significant difference in body perception impact was noted for patients who underwent a cystectomy (*p* = 0.033), although medians were similar between those with and without a cystectomy, 0.5 [0.33, 0.67] and 0.5 [0.38, 0.63} respectively.

### 3.6 Inter‐treatment comparisons

In addition to the above analyses, additional comparisons were made for the five themes within treatment types, such as between BCG, different types of chemotherapy, or radiotherapy. We did not find any difference in QOL measures for BCG therapy compared with other treatment modalities. Comparisons were also made between patients receiving radical radiotherapy against patients having a cystectomy. Finally, comparisons were made between the numbers of TURBT a patient had performed. In all of these comparisons, no significant differences in any of the themes were identified.

## 4 DISCUSSION

A deeper understanding of the lived experiences of patients with bladder cancer may provide insightful findings about the patient journey and highlight areas for service development and improvement. This study of data from 673 patients with at least T1 bladder cancer has integrated multiple datasets with the aim of providing this deeper insight. Significant differences between demographic groups in the impact of bladder cancer and its associated management were identified across five key themes. Although some of these findings may have been anticipated, others were unexpected and may demonstrate areas for future work focus.

First, it was identified that patients with long‐term health conditions reported significantly worse outcomes in all five themes explored. Although it may be anticipated that medical comorbidities may be associated with a higher impact on different domains of life, this does not distract from the fact that these patients are experiencing a greater impact on their lives compared with those without long‐term conditions. The results of this study do not identify specific areas of each theme where the greatest impact is noted; however, it may indicate that further work is required to look at the challenges faced by these patients, to see if increased support is required to address these differences.

Another significant finding, which may have been anticipated, is that patients with carer roles reported higher impact scores for lifestyle and ADL themes. It is well documented that individuals who undertake a caring role can be at risk of caregiver burden, which can be multifaceted and affect multiple domains of their lives, including schedule and lifestyle.^12^ Another potential explanation may be that these patients may not receive the same level of support from others when compared with those who are not caregivers. These results should prompt thoughts into whether patients with bladder cancer who are identified as caregivers should receive additional counselling on the potential impacts of bladder cancer on their lifestyle and ADLs, but also whether additional support could enable an improvement in these areas.

A significant difference in symptom impact scores was reported between sexes, with males reporting a higher level of impact. It is important to note that this theme was difficult to assess due to the inclusion of question relating to erectile dysfunction, which may have contributed to higher scores in males. Despite this, the results of this concur with a 2017 systematic review of patient experience studies in bladder cancer, which found that sexual concerns were a common symptom reported by patients.^5^ Catto et al. also support this, commenting that sexual dysfunction in men was found to score among the highest problem in a cross sectional survey of 3279 eligible patients with bladder cancer.^10^ Acknowledging this finding is important and indicates an important area for counselling male patients, especially for post‐cystectomy erectile dysfunction, where function may be improved by early pharmacological intervention.^13^

Smoking was associated with worse reported impact for lifestyle and psychological themed questions. Smoking is associated with an increased risk for bladder cancer^1^; hence, there are likely to be more patients with a smoking exposure history in a bladder cancer cohort compared with the general population. Because of the association with bladder cancer aetiology and worse lifestyle and psychological impact scores seen in smokers, specific counselling and smoking cessation advice for these patients may prove beneficial.

Further exploring the psychological impact of bladder cancer and its associated management, an analysis of the 2013 Wales Cancer Patient Experience Survey (WCPES) noted that psychological impact can be related to poor communication with patients, particularly during periods of treatment.^14^ This is corroborated by a 2012 co‐design experience study of lung and breast cancer patients, which found that patients with either lung cancer or breast cancer felt that they needed more information from treating teams.^15^ It may be beneficial for future studies to directly compare psychological impact in patients, with trials of different interventions aiming to reduce this impact. Guided by our study findings, it may be useful to evaluate whether patients with a smoking history or a long‐term health condition continue to demonstrate higher psychological impact levels and whether targeted interventions reduce this discrepancy.

Finally, results of particular interest come from the analyses of different management types. Our study found that other than differences in body perception and ADL themes for patients with a cystectomy compared with those without, there were no significant differences in any of the themes for within‐treatment comparisons (e.g. between different types of chemotherapy or radiotherapy strategies or number of TURBTs performed), between those with and without BCG, chemotherapy or between radical radiotherapy and cystectomy. The impact on body perception and ADLs for cystectomy patients compared with all other patients but not when compared with radical radiotherapy could reflect more T1 disease among those not treated with cystectomy, but staging data were insufficient to confirm this.

The comparisons made for patient experiences between different management types are relatively novel for this field given previous paucity in the data. For example, a previous systematic review by Edmondson et al. were unable to comment on this comparison as there was an over‐representation of patients undergoing surgical treatment in previous data sets used. Although our results come from a relatively small sample size, application of the data from the NCPES may aid with counselling patients about different management types.

### 4.1 Limitations

Although this study has strengths in utilising multiple datasets to gather an overview of patient‐lived experiences for bladder cancer, it does have several limitations, which must be acknowledged.

First, the study used data collected from the 2015 NCPES, a questionnaire sent to patients with a diagnosis of bladder cancer within the United Kingdom. By the nature of a voluntary questionnaire, there may be an element of selection bias as respondents may be of a particular demographic. Finally, the included sample size of 673 patients in this study is relatively small, particularly given the relatively high prevalence of the disease. These limitations combined may weaken the usability and representativeness of the dataset. To improve this, future studies could look to increase the sample sizes by incorporating further lived‐experience datasets, potentially including data from different countries.

Another potential limitation of this study was the way that the data were organised into the five themes and then subsequently analysed. The NCPES includes 99 questions, and organisation into themes was important to allow data comparison. However, identifying clear themes to organise the questions into were difficult, and it is possible that different researchers may have allocated the questions differently. In the future, this could be overcome with a consensus of themes described by more researchers or by analysing each NCPES question individually, although such an analysis would be prone to type 1 error. Despite this, analysing each individual question could provide a more detailed insight into specific aspects of the patient experience.

The omissions and missing data within the datasets used within this study may also limit the findings of the study; however, given the nature of the data collected, this may be difficult to overcome. In this study, geographical region of respondents was not disclosed due to relatively small numbers of patients in certain areas; however, if possible to obtain this information in future work, it could prove useful in highlighting centres with improved patient experiences to allow review and adoption of certain methods.

Although the above limitations restrict the application of the findings of this study, this study has utilised patient experience data to begin to explore why patients may experience different levels of impact on their lives. It may be pertinent to explore whether these trends extend across other cancer types to identify approaches to benefit a wider population. This links with a 2021 qualitative patient interview study of patients with biliary tract cancer, which reported that across all disease stages, the most salient symptoms included fatigue/lack of energy and impacts upon mobility and mental/emotional wellbeing.^16^

## 5 CONCLUSION

It has long been suspected that patients with bladder cancer face impacts on their lives due to the disease or management strategies; however, little evidence has been demonstrated from patient experience surveys. This study has identified both patient demographic factors and management strategies, which may have significant impact on patients' lives. Based on these findings, future work could be conducted to investigate how to address these factors and improve patient satisfaction in those with bladder cancer. The findings of this study also highlight a wide range of impacts on patient's lives, indicating a holistic care approach with involvement of the multidisciplinary team to support with different aspects of the patient's health and wellbeing.

It is hoped that the above findings can provide bladder cancer patients with greater understanding of their experiences and likely outcomes based on (but not limited to) their demographics and treatment type; in the long term, the data may be used to anticipate and minimise the impacts of treatment upon the lives of bladder cancer patients. The data from this study could also prove useful for service provision by both healthcare services and charities, as services could be developed to support those most affected. For example, a charity may be able to develop services to support those with carer responsibilities or other long‐term health conditions, with the aim of improving patient outcomes.

## AUTHOR CONTRIBUTIONS

All authors contributed equally to the design and manuscript. RK obtained the data from NCRAS. JI and SS analysed the data.

## CONFLICT OF INTEREST STATEMENT

R. K. is the chairman of Action Bladder Cancer UK charity. The remaining authors declare that they have no conflict of interest.
